# Prevalence of Discrimination, Abuse, and Harassment in Emergency Medicine Residency Training in the US

**DOI:** 10.1001/jamanetworkopen.2021.21706

**Published:** 2021-08-19

**Authors:** Michelle D. Lall, Karl Y. Bilimoria, Dave W. Lu, Tiannan Zhan, Melissa A. Barton, Yue-Yung Hu, Michael S. Beeson, James G. Adams, Lewis S. Nelson, Jill M. Baren

**Affiliations:** 1 Department of Emergency Medicine, Emory University, Atlanta, Georgia; 2 Department of Surgery, Northwestern University, Chicago, Illinois; 3 Department of Emergency Medicine, University of Washington, Seattle; 4 American Board of Emergency Medicine, East Lansing, Michigan; 5 Department of Emergency Medicine, Summa Health, Akron, Ohio; 6 Department of Emergency Medicine, Northwestern University, Chicago, Illinois; 7 Department of Emergency Medicine, Rutgers University, New Brunswick, New Jersey; 8 Department of Emergency Medicine, University of Pennsylvania, Philadelphia

## Abstract

**Importance:**

The prevalence of workplace mistreatment and its association with the well-being of emergency medicine (EM) residents is unclear. More information about the sources of mistreatment might encourage residency leadership to develop and implement more effective strategies to improve professional well-being not only during residency but also throughout the physician’s career.

**Objective:**

To examine the prevalence, types, and sources of perceived workplace mistreatment during training among EM residents in the US and the association between mistreatment and suicidal ideation.

**Design, Setting, and Participants:**

In this survey study conducted from February 25 to 29, 2020, all residents enrolled in EM residencies accredited by the Accreditation Council for Graduate Medical Education (ACGME) who participated in the 2020 American Board of Emergency Medicine computer-based In-training Examination were invited to participate. A multiple-choice, 35-item survey was administered after the examination asking residents to self-report the frequency, sources, and types of mistreatment experienced during residency training and whether they had suicidal thoughts.

**Main Outcomes and Measures:**

The types and frequency of workplace mistreatment and the sources of the mistreatment were identified, and rates of self-reported suicidality were obtained. Multivariable logistic regression models were used to examine resident and program characteristics associated with suicidal thoughts.

**Results:**

Of 8162 eligible EM residents, 7680 (94.1%) responded to at least 1 question on the survey; 6503 (79.7%) completed the survey in its entirety. A total of 243 ACGME-accredited residency programs participated, and 1 did not. The study cohort included 4768 male residents (62.1%), 2698 female residents (35.1%), 4919 non-Hispanic White residents (64.0%), 2620 residents from other racial/ethnic groups (Alaska Native, American Indian, Asian or Pacific Islander, African American, Mexican American, Native Hawaiian, Puerto Rican, other Hispanic, or mixed or other race) (34.1%), 483 residents who identified as lesbian, gay, bisexual, transgender, queer, or other (LGBTQ+) (6.3%), and 5951 residents who were married or in a relationship (77.5%). Of the total participants, 3463 (45.1%) reported exposure to some type of workplace mistreatment (eg, discrimination, abuse, or harassment) during the most recent academic year. A frequent source of mistreatment was identified as patients and/or patients’ families; 1234 respondents (58.7%) reported gender discrimination, 867 (67.5%) racial discrimination, 282 (85.2%) physical abuse, and 723 (69.1%) sexual harassment from patients and/or family members. Suicidal thoughts occurring during the past year were reported by 178 residents (2.5%), with similar prevalence by gender (108 men [2.4%]; 59 women [2.4%]) and race/ethnicity (113 non-Hispanic White residents [2.4%]; 65 residents from other racial/ethnic groups [2.7%]).

**Conclusions and Relevance:**

In this survey study, EM residents reported that workplace mistreatment occurred frequently. The findings suggest common sources of mistreatment for which educational interventions may be developed to help ensure resident wellness and career satisfaction.

## Key Points

**Question:**

What is the prevalence of workplace mistreatment and suicidal thoughts among emergency medicine residents?

**Findings:**

In this survey study of 7680 emergency medicine residents, 45.1% reported exposure to some type of workplace mistreatment (eg, discrimination, abuse, or harassment) and 2.5% reported having suicidal thoughts during the most recent academic year. The prevalence of workplace mistreatment and suicidal thoughts was similar by gender and race/ethnicity.

**Meaning:**

The findings suggest that educational interventions are warranted to reduce workplace mistreatment and ensure emergency medicine residents’ well-being during training.

## Teaser

This survey study examines the frequency, types, and sources of perceived workplace mistreatment and the prevalence of suicidal ideation among emergency medicine residents in the US.

## Introduction

Workplace mistreatment (eg, discrimination, harassment, abuse, and assault) and its institutional responses are associated with hostile work environments for physicians.^1,2,3,4^ The prevalence of workplace mistreatment has previously been shown to vary by gender and race/ethnicity, with the highest rates among women and racial/ethnic minority populations.^5,6^ Prior research has suggested that workplace mistreatment increases feelings of marginalization and may threaten a person’s sense of self, resulting in decreased job performance and productivity as well as increased stress, job dissatisfaction, negative workplace behaviors, and turnover.^5,7,8^ Workplace discrimination is associated with adverse physical and mental health, including increased rates of anxiety, depression, and cardiovascular disease.^7,8,9^

The current state of workplace mistreatment among emergency medicine (EM) residents remains unclear. Studies have documented workplace mistreatment in medicine, especially toward individuals with lower status in the medical workforce hierarchy.^1,4,6,10,11,12^ To our knowledge, the most recent comprehensive research on perceived mistreatment occurred more than 25 years ago in a study of 1774 EM residents.^13^ More recent work on harassment of EM residents has been limited by small sample sizes.^14,15^

Although there is a paucity of data on EM residents and their mistreatment experiences, a recent study of EM faculty revealed that women perceived significantly greater gender-based discrimination compared with men; 52.9% of female EM faculty and 26.2% of male EM faculty reported having experienced unwanted sexual behavior in the workplace at some point during their careers.^16^ Similarly, EM faculty from underrepresented racial/ethnic populations or sexual minority groups reported greater discrimination based on race/ethnicity or sexual orientation, respectively, compared with their colleagues.^16^

To understand the current prevalence of perceived workplace mistreatment among EM residents, a survey was administered to all residents enrolled in EM residencies accredited by the Accreditation Council for Graduate Medical Education (ACGME) to investigate the frequency, types, and sources of mistreatment. This study also examined the prevalence of suicidal ideation among EM residents in the US and assessed the associations between workplace mistreatment and suicidal ideation.

## Methods

### Study Settings and Participants

In this survey study conducted from February 25 to 29, 2020, a multiple-choice survey (eAppendix in the Supplement) was administered after the 2020 American Board of Emergency Medicine (ABEM) In-training Examination (ITE), an annual computer-based examination taken by residents training in ACGME-accredited EM residencies. In accordance with guiding principles of the American Association for Public Opinion Research (AAPOR), participants were provided a statement before the survey that informed them of the survey’s purpose and that it was voluntary, all data would be deidentified, program directors and department chairs would not have access to any responses, and participation would have no effect on the results of any ABEM examination. The Northwestern University institutional review board deemed this study as exempt because the responses were anonymous; voluntary participation in the study was considered consent to participate.

The survey used in this study was adapted from published surveys and therefore had substantial validity evidence for its use.^1,17,18,19,20^ All survey responses were recorded and stored in a highly secure server, deidentified by the ABEM, and submitted to Northwestern University’s Surgical Outcomes & Quality Improvement Center, which has relevant expertise in the data management and methodological approach that was used. Only residents enrolled in categorical, ACGME-accredited EM programs were included in the statistical analysis. Residents enrolled in other types of EM training such as combined EM programs (231 residents in 26 programs) and programs outside the US (54 residents in 4 programs) were excluded (Figure).

**Figure.**
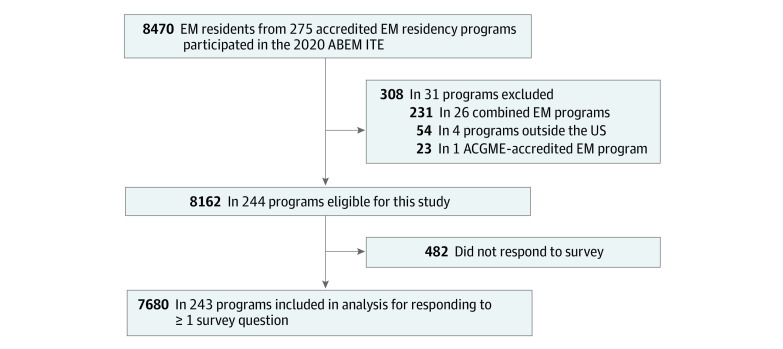
Flowchart of Residents and Programs Included in the Study ABEM ITE, American Board of Emergency Medicine In-training Examination; ACGME, Accreditation Council for Graduate Medical Education; EM, emergency medicine.

### Mistreatment Exposures

Respondents were asked to self-report^21^ the frequency of mistreatment since the beginning of the July 2019 academic year and to identify the primary source of mistreatment. Mistreatment types included discrimination based on self-identified gender, race/ethnicity, sexual orientation, and pregnancy or childcare status; physical, verbal, or emotional abuse; and sexual harassment. Frequency of mistreatment was categorized as never experienced, a few times a year, a few times a month, a few times a week, or every day. A single composite indicator was constructed for primary comparisons, representing the maximum reported frequency of any of the mistreatment exposures. Residents were then categorized by frequency of exposure to mistreatment into 1 of 3 groups: (1) no exposure, (2) exposures a few times per year, or (3) exposures a few times or more per month, including a few times per week or every day. Potential sources of mistreatment included patients and/or patients’ family members, attending physicians, other residents or fellows, administrators, nurses, and support staff.

### Suicidal Thoughts

Suicidal thoughts were assessed by asking residents at the end of the survey, “During the past 12 months, have you had thoughts of taking your own life?” After this question, participants were presented with a written statement urging them to seek their program director’s assistance if needed and were given the National Suicide Prevention Line contact information.

### Resident and Program Characteristics

Residents were queried about and self-reported gender, race/ethnicity, marital status, sexual orientation, and number of dependents (adult or child). Answer options were defined by the study investigators. The ABEM provided the clinical postgraduate year (PGY), categorized as PGY 1, PGY 2, PGY 3, or PGY 4, for each deidentified resident and program characteristics including program format (3-year or 4-year). Specific program information, such as name, city, and state, was also deidentified.

### Statistical Analysis

To compare the unadjusted prevalence of dichotomized mistreatment exposures and outcomes stratified by gender and race/ethnicity, we used the Pearson χ^2^ test with α = 0.01. Multivariable logistic regression models were used to examine all available resident and program characteristics associated with suicidal thoughts both including and excluding the composite mistreatment exposure. Clustered robust standard error adjustment was applied to all models to account for clustering within EM residencies. In each model, residents with missing responses for any of the variables and outcomes were excluded from the data analysis (exclusion rate, 10.0%-12.5%). Odds ratios with Bonferroni-corrected 99% CIs are reported. Significance was set at *P* < .05 using a 2-tailed test. All statistical analyses were performed using R, version 3.6.2 (R Project for Statistical Computing).

## Results

### Survey Response

Altogether, 243 ACGME-accredited EM residency programs participated in the ITE, and 1 (23 residents) did not. Of the 8162 eligible EM residents, 7680 (94.1%) responded to at least 1 question on the survey; 6503 (79.7%) completed the survey in its entirety. The study cohort included 4768 male residents (62.1%), 2698 female residents (35.1%), 4919 non-Hispanic White residents (64.0%), and 2620 residents (34.1%) from other racial/ethnic groups (African American, 282 [3.7%]; Asian or Pacific Islander, 1042 [13.6%]; Hispanic or Latino, 329 [4.3%]; Native American or Alaska Native, 33 [0.4%]; and mixed race or other, 934 [12.2%]). A total of 483 residents (6.3%) identified as lesbian, gay, bisexual, transgender, queer, or other (LGBTQ+), and 5951 (77.5%) were married or in a relationship (Table 1). Of the total participants, 3463 (45.1%) reported exposure to some type of workplace mistreatment (eg, discrimination, abuse, or harassment) during the most recent academic year. A frequent source of mistreatment was identified as patients and/or patients’ families; 1234 respondents (58.7%) reported gender discrimination, 867 (67.5%) racial discrimination, 282 (85.2%) physical abuse, and 723 (69.1%) sexual harassment from patients and/or family members.

**Table 1.** Study Population Demographics, Stratified by Self-reported Gender and Race/Ethnicity

Characteristic	Respondents, No. (%)				
	Overall (N = 7680)	Sex		Race/ethnicity	
		Men (n = 4768)	Women (n = 2698)	White (n = 4919)	Racial/ethnic minority groups (n = 2620)
Gender					
Male	4768 (62.1)	NA	NA	3170 (64.4)	1531 (58.4)
Female	2698 (35.1)	NA	NA	1687 (34.3)	984 (37.6)
Unknown^a^	214 (2.8)	NA	NA	62 (1.3)	105 (4.0)
Race/ethnicity					
Non-Hispanic White	4919 (64.0)	3170 (66.5)	1687 (62.5)	4919 (100.0)	NA
African American	282 (3.7)	145 (3.0)	135 (5.0)	NA	282 (10.8)
Asian or Pacific Islander	1042 (13.6)	631 (13.2)	398 (14.8)	NA	1042 (39.8)
Hispanic or Latino American	329 (4.3)	202 (4.2)	124 (4.6)	NA	329 (12.6)
Native American or Alaska Native	33 (0.4)	22 (0.5)	6 (0.2)	NA	33 (1.3)
Other or mixed^b^	934 (12.2)	531 (11.1)	321 (11.9)	NA	934 (35.6)
Unknown	141 (1.8)	67 (1.4)	27 (1.0)	NA	NA
Sexual orientation					
Not LGBTQ+	6900 (89.8)	4389 (92.1)	2428 (90.0)	4560 (92.7)	2324 (88.7)
LGBTQ+	483 (6.3)	259 (5.4)	205 (7.6)	307 (6.2)	176 (6.7)
Other	132 (1.7)	39 (0.8)	32 (1.2)	34 (0.7)	98 (3.7)
Unknown	165 (2.1)	81 (1.7)	33 (1.2)	18 (0.4)	22 (0.8)
Clinical postgraduate year					
1	2522 (32.8)	1580 (33.1)	877 (32.5)	1571 (31.9)	897 (34.2)
2	2335 (30.4)	1462 (30.7)	809 (30.0)	1496 (30.4)	800 (30.5)
3	2227 (29.0)	1388 (29.1)	776 (28.8)	1471 (29.9)	722 (27.6)
4	596 (7.8)	338 (7.1)	236 (8.7)	381 (7.7)	201 (7.7)
Residency format					
PGY 1-3	5300 (69.0)	3374 (70.8)	1780 (66.0)	3489 (70.9)	1720 (65.6)
PGY 1-4	2380 (31.0)	1394 (29.2)	918 (34.0)	1430 (29.1)	900 (34.4)
Program size^c^					
Quartile 1	1334 (17.4)	882 (18.5)	415 (15.4)	856 (17.4)	450 (17.2)
Quartile 2	1391 (18.1)	891 (18.7)	463 (17.2)	957 (19.5)	414 (15.8)
Quartile 3	2222 (28.9)	1365 (28.6)	800 (29.7)	1500 (30.5)	688 (26.3)
Quartile 4	2733 (35.6)	1630 (34.2)	1020 (37.8)	1606 (32.6)	1068 (40.8)
Residency region^d^					
1	1124 (14.6)	680 (14.3)	408 (15.1)	686 (13.9)	417 (15.9)
2	752 (9.8)	481 (10.1)	254 (9.4)	521 (10.6)	220 (8.4)
3	767 (10.0)	493 (10.3)	254 (9.4)	533 (10.8)	227 (8.7)
4	1088 (14.2)	689 (14.5)	364 (13.5)	670 (13.6)	399 (15.2)
5	1311 (17.1)	845 (17.7)	431 (16.0)	963 (19.6)	328 (12.5)
6	1156 (15.1)	686 (14.4)	444 (16.5)	770 (15.7)	366 (14.0)
7	1482 (19.3)	894 (18.8)	543 (20.1)	776 (15.8)	663 (25.3)
Relationship status					
Married or in a relationship	5951 (77.5)	3837 (80.5)	1980 (73.4)	4010 (81.5)	1904 (72.7)
Not in a relationship	1511 (19.7)	833 (17.5)	649 (24.1)	827 (16.8)	675 (25.8)
Divorced or widowed	105 (1.4)	51 (1.1)	45 (1.7)	71 (1.4)	34 (1.3)
Unknown	113 (1.5)	47 (1.0)	24 (0.9)	11 (0.2)	7 (0.3)
Pregnancy or childcare					
Yes	1544 (20.1)	1158 (24.3)	335 (12.4)	1161 (23.6)	373 (14.2)
No	5972 (77.8)	3531 (74.1)	2330 (86.4)	3740 (76.0)	2216 (84.6)
Unknown	164 (2.1)	79 (1.7)	33 (1.2)	18 (0.4)	31 (1.2)
Other dependent(s)					
Yes	287 (3.7)	170 (3.6)	98 (3.6)	154 (3.1)	132 (5.0)
No	7190 (93.6)	4495 (94.3)	2555 (94.7)	4732 (96.2)	2438 (93.1)
Unknown	203 (2.6)	103 (2.2)	45 (1.7)	33 (0.7)	50 (1.9)

Abbreviations: LGBTQ+, lesbian, gay, bisexual, transgender, queer, or other; NA, not applicable; PGY, postgraduate year.

^a^ Includes residents who selected “prefer not to answer” and those who skipped this question.

^b^ Other included those who did not identify as one of the racial/ethnic groups listed on the survey as a response choice.

^c^ Quartile 1 was less than 25 residents; quartile 2, 25 to 32 residents; quartile 3, 33 to 42 residents; and quartile 4, more than 42 residents.

^d^ Residency regions were the Medical Student Section Regions defined by the American Medical Association.

### Mistreatment

As shown in Table 2, gender discrimination was reported by 2104 residents (29.5%; 1635 women [65.2%]; 407 men [9.1%]; *P* < .001), with the most common sources for both men and women being patients or patients’ family members (1027 women [62.8%]; 184 men [45.2%]) followed by nurses or staff (331 women [20.2%]; 59 men [14.5%]) (Table 3). Racial discrimination was reported by 1284 residents (18.0%), including 371 White residents (7.9%) and 907 residents from other racial/ethnic groups (37.6%) (*P* < .001). In addition, 248 residents (10.3%) from other racial/ethnic groups reported a frequency of exposure to racial discrimination as a few times per month or more. The most common source of racial discrimination reported was patients or patients’ family members (224 White residents [60.4%]; 639 residents from other racial/ethnic groups [70.5%]).

**Table 2.** Frequency of Resident Mistreatment Among the Study Population by Gender and Race/Ethnicity

Type of mistreatment	Residents experiencing mistreatment, No. (%)			*P* value	Residents experiencing mistreatment, No. (%)		*P* value
	Overall (N = 7680)	Sex			Race/ethnicity		
		Men (n = 4768)	Women (n = 2698)		White (n = 4919)	Not White (n = 2620)	
Discrimination based on gender							
Overall	2104 (29.5)	407 (9.1)	1635 (65.2)	<.001	1330 (28.2)	764 (31.7)	.002
A few times per year	1342 (18.8)	358 (8.0)	952 (37.9)	NA	855 (18.2)	480 (19.9)	NA
A few times per month or more frequently	762 (10.7)	49 (1.1)	683 (27.2)	NA	475 (10.1)	284 (11.8)	NA
Discrimination based on race/ethnicity							
Overall	1284 (18.0)	695 (15.5)	548 (21.9)	<.001	371 (7.9)	907 (37.6)	<.001
A few times per year	970 (13.6)	543 (12.1)	407 (16.2)	NA	307 (6.5)	659 (27.3)	NA
A few times per month or more frequently	314 (4.4)	152 (3.4)	141 (5.6)	NA	64 (1.4)	248 (10.3)	NA
Discrimination based on sexual orientation							
Overall	220 (3.1)	139 (3.1)	68 (2.7)	.41	142 (3.0)	77 (3.2)	.71
A few times per year	173 (2.4)	116 (2.6)	51 (2.0)	NA	118 (2.5)	54 (2.3)	NA
A few times per month or more frequently	47 (0.7)	23 (0.5)	17 (0.7)	NA	24 (0.5)	23 (1.0)	NA
Discrimination based on pregnancy or childcare status							
Overall	365 (5.1)	79 (1.8)	270 (10.8)	<.001	264 (5.6)	98 (4.1)	.007
A few times per year	283 (4.0)	55 (1.2)	221 (8.8)	NA	214 (4.6)	68 (2.8)	NA
A few times per month or more frequently	82 (1.2)	24 (0.5)	49 (2.0)	NA	50 (1.1)	30 (1.3)	NA
Any discrimination^a^							
Overall	2665 (34.7)	921 (19.3)	1677 (62.2)	<.001	1489 (30.3)	1165 (44.5)	<.001
A few times per year	1717 (24.2)	730 (16.3)	956 (38.4)	NA	962 (20.5)	748 (31.3)	NA
A few times per month or more frequently	919 (12.9)	184 (4.1)	704 (28.3)	NA	516 (11.0)	400 (16.8)	NA
Verbal or emotional abuse							
Overall	2069 (29.0)	1212 (27.0)	806 (32.2)	<.001	1269 (27.0)	790 (32.9)	<.001
A few times per year	1436 (20.1)	851 (19.0)	556 (22.2)	NA	894 (19.0)	537 (22.3)	NA
A few times per month or more frequently	633 (8.9)	361 (8.0)	250 (10.0)	NA	375 (8.0)	253 (10.5)	NA
Physical abuse							
Overall	331 (4.6)	207 (4.6)	111 (4.4)	.77	201 (4.3)	126 (5.2)	.07
A few times per year	285 (4.0)	171 (3.8)	104 (4.2)	NA	175 (3.7)	107 (4.5)	NA
A few times per month or more frequently	46 (0.6)	36 (0.8)	7 (0.3)	NA	26 (0.6)	19 (0.8)	NA
Any abuse^b^							
Overall	2086 (27.2)	1220 (25.6)	814 (30.2)	<.001	1282 (26.1)	794 (30.3)	<.001
A few times per year	1449 (20.4)	857 (19.1)	562 (22.5)	NA	904 (19.2)	540 (22.5)	NA
A few times per month or more frequently	624 (8.8)	357 (8.0)	246 (9.8)	NA	372 (7.9)	247 (10.3)	NA
Sexual harassment							
Overall	1047 (14.7)	294 (6.5)	721 (28.8)	<.001	695 (14.8)	347 (14.4)	.72
A few times per year	846 (11.9)	264 (5.9)	556 (22.2)	NA	557 (11.8)	285 (11.9)	NA
A few times per month or more frequently	201 (2.8)	30 (0.7)	165 (6.6)	NA	138 (2.9)	62 (2.6)	NA
Any exposure to discrimination, abuse, or harassment							
Overall	3463 (45.1)	1604 (33.6)	1777 (65.9)	<.001	2079 (42.3)	1368 (52.2)	<.001
A few times per year	2117 (29.9)	1117 (25.0)	964 (38.8)	NA	1297 (27.7)	811 (34.0)	NA
A few times per month or more frequently	1296 (18.3)	469 (10.5)	788 (31.7)	NA	761 (16.3)	529 (22.2)	NA
Suicidal thoughts	178 (2.5)	108 (2.4)	59 (2.4)	.96	113 (2.4)	65 (2.7)	.51

Abbreviation: NA, not applicable.

^a^ Discrimination based on gender, race, sexual orientation, or pregnancy or childcare status.

^b^ Verbal, emotional, or physical abuse.

**Table 3.** Sources of Mistreatment, by Relevant Subgroups

Type of mistreatment	Residents experiencing mistreatment, No. (%)						
	Total, No.	Source of mistreatment					
		Patients or family	Attending physicians	Administration	Other residents or fellows	Nurses or staff	No response
Gender discrimination							
All	2104	1234 (58.7)	175 (8.3)	16 (0.8)	89 (4.2)	406 (19.3)	184 (8.7)
Men	407	184 (45.2)	45 (11.1)	9 (2.2)	34 (8.4)	59 (14.5)	76 (18.7)
Women	1635	1027 (62.8)	124 (7.6)	6 (0.4)	53 (3.2)	331 (20.2)	94 (5.7)
Discrimination based on sexual orientation							
All	220	98 (44.5)	19 (8.6)	4 (1.8)	30 (13.6)	14 (6.4)	55 (25.0)
Not LGBTQ+	89	25 (28.1)	4 (4.5)	2 (2.2)	12 (13.5)	8 (9.0)	38 (42.7)
LGBTQ+	130	73 (56.2)	15 (11.5)	2 (1.5)	18 (13.8)	6 (4.6)	16 (12.3)
Discrimination based on pregnancy or childcare status							
All	365	53 (14.5)	97 (26.6)	28 (7.7)	82 (22.5)	23 (6.3)	82 (22.5)
Not associated with parenting	177	34 (19.2)	41 (23.2)	11 (6.2)	25 (14.1)	14 (7.9)	52 (29.4)
Associated with parenting	186	18 (9.7)	55 (29.6)	17 (9.1)	57 (30.6)	9 (4.8)	30 (16.1)
Discrimination based on race/ethnicity							
All	1284	867 (67.5)	60 (4.7)	18 (1.4)	61 (4.8)	107 (8.3)	171 (13.3)
White	371	224 (60.4)	16 (4.3)	9 (2.4)	22 (5.9)	23 (6.2)	77 (20.8)
Not White	907	639 (70.5)	44 (4.9)	9 (1.0)	38 (4.2)	84 (9.3)	93 (10.3)
Verbal or emotional abuse							
All	2069	1231 (59.5)	388 (18.8)	15 (0.7)	125 (6.0)	141 (6.8)	169 (8.2)
Men	1212	702 (57.9)	259 (21.4)	9 (0.7)	82 (6.8)	81 (6.7)	79 (6.5)
Women	806	503 (62.4)	120 (14.9)	6 (0.7)	42 (5.2)	55 (6.8)	80 (9.9)
Physical abuse							
All	331	282 (85.2)	6 (1.8)	2 (0.6)	3 (0.9)	3 (0.9)	35 (10.6)
Men	207	182 (87.9)	4 (1.9)	2 (1.0)	0 (0.0)	1 (0.5)	18 (8.7)
Women	111	95 (85.6)	2 (1.8)	0 (0.0)	2 (1.8)	1 (0.9)	11 (9.9)
Sexual harassment							
All	1047	723 (69.1)	52 (5.0)	6 (0.6)	33 (3.2)	127 (12.1)	106 (10.1)
Men	294	181 (61.6)	5 (1.7)	2 (0.7)	7 (2.4)	64 (21.8)	35 (11.9)
Women	721	525 (72.8)	46 (6.4)	4 (0.6)	23 (3.2)	61 (8.5)	62 (8.6)

Abbreviation: LGBTQ+, lesbian, gay, bisexual, transgender, queer, or other.

Discrimination based on sexual orientation or gender identity was reported by 220 residents (3.1%). Most LGBTQ+ residents (73 of 130 [56.2%]) who reported discrimination identified patients or patients’ families as the primary source, whereas 18 (13.8%) identified other residents as the primary source.

Discrimination based on pregnancy or childcare status was reported by 365 residents (5.1%), including 270 women (10.8%) and 79 men (1.8%) (*P* < .001). Among the 186 residents who self-identified as parents (defined as respondents who reported having children younger than 18 years or respondents who were or whose partner was pregnant, adopting, or expecting a child from July 2019 to the date of the ABEM ITE examination), 57 (30.6%) identified other residents as the primary source of discrimination and 55 (29.6%) identified attending physicians.

Sexual harassment was reported by 1047 residents (14.7%), including 721 women (28.8%) and 294 men (6.5%) (*P* < .001). The main source was identified as patients and/or patients’ family members (525 women [72.8%]; 181 men [61.6%]), followed by nurses and staff (64 men [21.8%]; 61 women [8.5%]).

Overall, 2069 residents (29.0%) reported verbal or emotional abuse, including 806 women (32.2%) and 1212 men (27.0%) (*P* < .001). The most common sources were patients or patients’ family members (503 women [62.4%]; 702 men [57.9%]), followed by attending physicians (259 men [21.4%]; 120 women [14.9%]). Physical abuse was reported by 331 residents (4.6%) and was primarily attributed to patients or patients’ families.

### Suicidal Thoughts and Associated Factors

Suicidal thoughts were reported by 178 residents (2.5%), with similar prevalence by gender (108 men [2.4%]; 59 women [2.4%]) and race/ethnicity (113 non-Hispanic White individuals [2.4%]; 65 residents from other racial/ethnic groups [2.7%]).

In the adjusted models for suicidal thoughts, the prevalence was greater for residents who identified as LGBTQ+ (odds ratio [OR], 2.04; 99% CI, 1.04-3.99) (Table 4). Residents who were divorced or widowed had greater odds of reporting suicidal thoughts (OR, 3.36; 99% CI, 1.06-10.70) compared with residents who were married or in a relationship.

**Table 4.** Odds of Experiencing Suicidal Thoughts by Resident Characteristic, Adjusted for PGY, Relationship Status, Program Size, and Program Location

Characteristic	Residents with suicidal thoughts, No. (%) (N = 6721)	Odds ratio (99% CI)	
		Excluding mistreatment measures	Including mistreatment measures
Overall	159 (2.4)	NA	NA
Gender			
Male	104 (2.4)	1 [Reference]	1 [Reference]
Female	55 (2.3)	0.90 (0.58-1.40)	0.54 (0.34-0.86)
Race/ethnicity			
White	102 (2.3)	1 [Reference]	1 [Reference]
Racial/ethnic minority groups	57 (2.6)	1.15 (0.73-1.79)	0.99 (0.62-1.58)
Clinical PGY			
1	45 (2.0)	1 [Reference]	1 [Reference]
2	60 (2.9)	1.36 (0.84-2.21)	1.22 (0.72-2.06)
3-4	54 (2.2)	0.99 (0.56-1.75)	0.83 (0.46-1.49)
Residency format			
PGY 1-3	108 (2.3)	1 [Reference]	1 [Reference]
PGY 1-4	51 (2.5)	0.94 (0.57-1.55)	0.89 (0.52-1.51)
Sexual orientation			
Not LGBTQ+	136 (2.2)	1 [Reference]	1 [Reference]
LGBTQ+	23 (4.7)	2.41 (1.29-4.50)	2.04 (1.04-3.99)
Relationship status			
Married or in a relationship	116 (2.2)	1 [Reference]	1 [Reference]
No relationship	37 (2.8)	1.34 (0.92-1.96)	1.23 (0.75-2.01)
Divorced or widowed	6 (7.1)	4.28 (1.91-9.62)	3.36 (1.06-10.70)
Pregnancy or childcare			
Yes	126 (2.4)	1.10 (0.59-2.04)	1.11 (0.59-2.09)
No	33 (2.4)	1 [Reference]	1 [Reference]
Other dependent(s)			
Yes	150 (2.3)	1.62 (0.62-4.23)	1.70 (0.65-4.48)
No	9 (3.8)	1 [Reference]	1 [Reference]
Program size^a^			
Quartile 1	26 (2.2)	1 [Reference]	1 [Reference]
Quartile 2	17 (1.4)	0.53 (0.25-1.14)	0.59 (0.27-1.27)
Quartile 3	58 (2.9)	1.16 (0.64-2.10)	1.24 (0.68-2.27)
Quartile 4	58 (2.5)	1.02 (0.55-1.91)	0.99 (0.51-1.91)
Residency region^b^			
1	28 (2.9)	1 [Reference]	1 [Reference]
2	22 (3.2)	1.12 (0.50-2.49)	1.18 (0.53-2.66)
3	16 (2.4)	0.77 (0.36-1.64)	0.93 (0.44-1.98)
4	14 (1.5)	0.53 (0.22-1.25)	0.52 (0.23-1.21)
5	26 (2.2)	0.83 (0.40-1.72)	0.86 (0.40-1.82)
6	26 (2.5)	0.81 (0.37-1.74)	0.88 (0.40-1.92)
7	27 (2.1)	0.74 (0.37-1.48)	0.65 (0.31-1.35)
Frequency of mistreatment			
Never	47 (1.3)	NA	1 [Reference]
A few times per year	42 (2.1)	NA	1.69 (0.90-3.15)
A few times per month or more	70 (5.8)	NA	5.83 (3.70-9.20)

Abbreviations: LGBTQ+, lesbian, gay, bisexual, transgender, queer, or other; NA, not applicable; PGY, postgraduate year.

^a^ Quartile 1 was less than 25 residents; quartile 2, 25 to 32 residents; quartile 3, 33 to 42 residents; and quartile 4, more than 42 residents.

^b^ Residency regions were the Medical Student Section Regions defined by the American Medical Association.

An association was observed between experiencing mistreatment at least a few times per month and having suicidal thoughts (OR, 5.83; 99% CI, 3.70-9.20). Residents who had never experienced mistreatment or had experienced it only a few times a year did not report having suicidal thoughts. After adjusting for mistreatment exposures, women were less likely than men to report suicidal thoughts (OR, 0.54; 99% CI, 0.34-0.86).

## Discussion

In this comprehensive survey study, mistreatment of EM residents based on gender, race/ethnicity, and sexual orientation was more common among women, residents from racial/ethnic minority populations, and residents identifying as LGBTQ+, respectively. Discrimination based on pregnancy and childcare status was also more common among women than among men. Although expectant fathers were not separately counted, they were included because the survey did not specifically state the gender of the expectant parent but rather the childcare status of the resident. Workplace mistreatment was associated with suicidal thoughts; however, after adjusting for mistreatment, women were less likely than men to report suicidal ideation. Given the high response rates to the survey, the investigators were confident that the study population represented the general population of EM residents.

Working in the emergency department is associated with a higher risk for violence, abuse, and harassment because of its high-stress, fast-paced environment^15,22,23^ and exposure to certain patient populations who seek emergency care (eg, patients with psychiatric diagnoses, intoxicated patients,^24^ and incarcerated patients^22^). Workplace violence is associated with an increase in the likelihood of burnout, depression, and posttraumatic stress disorder in physicians.^25^ In this study, 45.1% of all EM residents reported some form of mistreatment, which was less than the 98% of EM residents who reported at least 1 occurrence of abuse or harassment in a 1993 survey^13^ in which many residents noted that abuse from patients was “just part of the job.”^13^

Broad cultural changes and movements within medicine and society at large (including TIME’S UP Healthcare, #MeToo, #HeForShe, White Coats for Black Lives, and Black Lives Matter) have raised awareness of the detriment of systemic racism^26^ and the benefits of diversity and equity^27^ and may be associated with decreased reporting of discrimination in the workplace during the past 3 decades.^28^ It is possible that respondents to the current study’s survey may have underreported mistreatment owing to differences in study design. In contrast to the 1993 study,^13^ discrimination, abuse, and harassment were intentionally not defined in this study but were left open to respondent interpretation. Whether changing definitions of mistreatment affects reporting rates is unknown, and this is an area for future research.^29^

Identifying sources of mistreatment is important because it helps inform how institutions and individuals should intervene. Consistent with the findings of the current study, patients or their families have remained the most frequent source of all forms of mistreatment in the past 27 years.^13^ However, respondents to this survey indicated that discrimination based on pregnancy and childcare status was perpetrated most often by attending physicians and other residents.

Women reported higher levels of nearly all forms of mistreatment compared with men, with most of the reported gender-based mistreatment originating from patients and their families. The second most likely source of gender-based mistreatment was nurses and staff. Gender bias, discrimination, and sexual harassment in medicine have deleterious consequences for women physicians’ careers and well-being.^10,12,30,31,32,33,34,35,36,37,38,39^

Mistreatment of primarily women in medicine may be associated with fewer women seeking EM careers, and the comparative underrepresentation of women in EM may be associated with their greater mistreatment. More than 50% of medical student matriculants are female; however, female physicians receive less compensation, opportunity for advancement, scholarship, and recognition compared with men,^12,32,38^ and EM is no exception. Women represent approximately 30% of emergency physicians, and this percentage has been consistent for the past decade.^40^ A study by Madsen et al^12^ showed that salaries for women in academic EM were at least $19 000 less than those for their male peers. Women in EM represent a minority of professors and department chairs.^38,41^ Women are also less likely to be first authors of scholarly work and less likely to be part of editorial boards for the major specialty journals.^42,43^ The findings of this study are consistent with prior work showing gender discrimination and mistreatment in academic medicine.^44^ Institutional policies that recognize bias, destigmatize reporting, and promote education regarding inclusivity may be helpful.

Emergency medicine physicians are predominantly White individuals, and there are comparatively few physicians in the specialty who identify as underrepresented in medicine.^45,46,47,48^ In the cohort of EM residents in the present study, most residents (64.0%) identified as non-Hispanic White, and few residents identified as African American (3.7%) or Hispanic or Latino American (4.3%). Racial bias and discrimination are present in medicine, and subtle forms of bias and discrimination may begin in medical school.^45,49^ Resident physicians have reported experiencing many microaggressions daily and bias during their training.^50^ The findings of this study are consistent with prior work in which individuals who identified as underrepresented in medicine were found to have experienced higher rates of discrimination than their peers who did not identify as underrepresented in medicine.^30,51,52^

Discrimination by patients toward physicians also occurs on the basis of sexual orientation and gender identity.^53,54^ In this study, discrimination based on sexual orientation or gender identity was reported by 3.1% of residents. Of the residents who identified as LGBTQ+ and who reported discrimination, 56.2% identified patients or families as the primary source and 13.8% identified the primary source as other residents.

### Suicidal Thoughts

In this study, 2.5% of the EM residents reported having suicidal thoughts during the July 2019 to February 2020 academic year (Table 2), which was lower than the rate reported in previous studies.^1,55,56,57,58,59^ Similar to the findings of other work,^60,61,62,63^ residents in this study who identified as LGBTQ+ and those who were divorced or widowed reported higher rates of suicidal thoughts. Physician suicide should be equated to a “never event” (an event that is preventable and should never occur), highlighting the need for even greater suicide prevention initiatives. Compared with the general population, physicians have a higher likelihood of suicide, and female physicians are at greater risk than male physicians.^64,65^ In this study, there was a significant association between the reported frequency of mistreatment and suicidal thoughts. After adjusting for mistreatment, women were less likely to report suicidal thoughts. The results suggest that the higher prevalence of mistreatment experienced by women in medicine may be 1 factor associated with the higher rates of suicide among female physicians. Systemic interventions appear to be needed to address workplace mistreatment. Leaders, peers, and other hospital colleagues may be bystanders, perhaps inadvertently, to workplace mistreatment. Health care systems, hospitals, and department and residency program leaders should consider training interventions to empower bystanders to intervene and to cultivate workplace norms that prohibit workplace mistreatment.^66^ An additional strategy is to provide cultural competency training to all emergency department staff with the goal of increasing collective knowledge about marginalized groups (women and individuals who are underrepresented in medicine or LGBTQ+) that are at increased risk of experiencing workplace mistreatment.^67,68^ This increase in knowledge and subsequent self-awareness may create a more open, safe, and supportive workplace for EM residents.

There is a paucity of data on resident mistreatment. A survey^1^ regarding mistreatment and suicidal ideation was conducted in 2018 among general surgery residents, and compared with findings of that study, EM residents in this study reported lower rates of suicidal ideation (EM vs surgery: 2.5% vs 4.5%) but higher rates of sexual harassment (EM vs surgery: 28.8% vs 19.9%). Both EM and general surgery are predominantly male specialties, and future work should compare these results with those from other specialties.

Residents in many programs are likely able to train in environments without frequent exposures to discrimination, harassment, or abuse. Mistreatment should never be considered an acceptable occurrence in any training program. This study was not able to examine which systems, programs, or cultural factors were associated with lower rates of mistreatment in some institutions and higher rates in others. Qualitative studies of residents and program leaders from institutions with varying levels of reported mistreatment may help address this question.

### Limitations

This study has limitations. Although all data were deidentified, it is conceivable that concern regarding identification of respondents may have resulted in underreporting of harassment owing to fear of retaliation. Social-desirability bias may have contributed to underreporting to portray strength and stamina as an emergency physician. Although unlikely, it is also conceivable that overreporting might have occurred owing to the anonymous nature of the survey. No option for discrimination based on gender identity was provided in the survey. As a result, residents identifying as nonbinary may have responded based on their gender or sexual orientation, limiting the degree to which discrimination based on gender identity in this study could be assessed.

The consequences of administering the survey after the 4.5-hour, 225 multiple-choice question ITE are also unknown. This type of approach has a precedence because a similar survey on resident mistreatment was previously conducted by the American Board of Surgery.^1^ Given the length and duration of the ITE, residents may not have been inclined to participate in the survey or to complete it in its entirety. Nonetheless, the survey response rate was high. Examination-related anxiety may have been associated with reporting more negative feelings. On the contrary, relief from completing the examination may have diminished negative feelings. In addition, there may be inherent recall bias owing to the time lapse between the start of the academic year and the ITE administration in the subsequent February.

In the current study, mistreatment definitions were not provided but instead were open to the resident’s interpretation. Therefore, the number of reported mistreatment occurrences may have been lower than the actual occurrences. As reported previously, many EM clinicians consider verbal abuse, insults, and other derogatory behavior to be normal and just a part of the job.^13^ As a result, emergency department violence, abuse, and harassment are likely to be underreported.^23,69^

## Conclusions

In this survey study, EM residents reported commonly experiencing workplace mistreatment, and experiences of mistreatment were associated with suicidality. Identifying and promoting best practices to minimize workplace mistreatment during residency may help optimize the professional career experience and improve the personal and professional well-being of physicians throughout their lives.

## Notes

Supplement. **eAppendix.** Survey
