# Supplementary material for: Prevalence of Discrimination, Abuse, and Harassment in Emergency Medicine Residency Training in the US
Source: JAMA Netw Open. 2021 Aug 19;4(8):e2121706. doi: 10.1001/jamanetworkopen.2021.21706 (PMC8377562; doi:10.1001/jamanetworkopen.2021.21706)
Supplement: Supplement. — eAppendix. Survey [file jamanetwopen-e2121706-s001.pdf]

Supplementary Online Content

Lall MD, Bilimoria KY, Lu DW, et al. Prevalence of discrimination, abuse, and harassment in emergency medicine residency training in the US. *JAMA Netw Open*. 2021;4(8):e2121706. doi:10.1001/jamanetworkopen.2021.21706

eAppendix. Survey

This supplementary material has been provided by the authors to give readers additional information about their work.

© 2021 Lall MD et al. *JAMA Network Open*.

eAppendix. Survey

Survey Preamble

Please complete the following survey to share your thoughts about your residency experience. The information will be used to inform future research and policy decisions within the specialty of Emergency Medicine. It is estimated that it will take approximately 10 minutes to complete the survey.

Any reported data will be aggregated and de-identified with respect to training program and individual responses. Your program director and Chair will **not** have access to your individual responses or your program's responses. Survey results will have **no** effect on your performance on any ABEM examination.

Demographics

1. I identify myself as:
    a. Male
    b. Female
    c. Prefer not to respond

2. The following describes me:
    a. Married
    b. Not married but in a relationship
    c. Not married and not in a relationship
    d. Divorced
    e. Widowed

3. The following best describes my ethnicity:
    a. Alaskan native
    b. American Indian
    c. Asian or Pacific Islander
    d. African American
    e. Mexican American
    f. Native Hawaiian
    g. Puerto Rican
    h. Caucasian (non-Hispanic)
    i. Other Hispanic
    j. Mixed Race
    k. Other

4. I identify myself as:
    a. Heterosexual
    b. Gay
    c. Lesbian
    d. Bisexual
    e. Transgender
    f. Queer
    g. Other

5. Do you have children under age 18 *OR* are you or your partner pregnant/adopting/expecting a child this academic year?
    a. Yes
    b. No

© 2021 Lall MD et al. *JAMA Network Open*.

6. Do you care for a dependent <u>other</u> than a child under age 18 *OR* do you or your partner expect to care for a dependent this academic year (e.g., elderly parent, adult ill relative, adult child with special needs, etc.)?
    a. Yes
    b. No

7. Since the beginning of this academic year (July 2019), have you experienced the following during residency?

	Never experienced	A few times a year	A few times a month	A few times a week	Every day
a. Discrimination based on gender					
b. Discrimination based on race/ethnicity					
c. Discrimination based on sexual orientation					
d. Discrimination based on past, present, or expected pregnancy or childcare status					
e. Physical abuse					
f. Verbal or emotional abuse					
g. Sexual harassment					

8. Based on your answers from the previous question (question 7), from whom did you experience the discrimination, abuse or harassment during residency? Please answer for only those categories that you experienced discrimination, abuse or harassment; categories that you marked as "never" you may disregard.

	Never experienced	Patient(s) and/or patient's family members	Attending(s)	Administrator(s)	Colleagues (e.g., other residents, fellows)	Nurses, ancillary staff (radiology/lab technicians, clerical, IT personnel, etc.)
a. Discrimination based on gender						
b. Discrimination based on race/ethnicity						
c. Discrimination based on sexual orientation						

© 2021 Lall MD et al. *JAMA Network Open*.

d. Discrimination based on past, present, or expected pregnancy or child care status						
e. Physical abuse						
f. Verbal or emotional abuse						
g. Sexual harassment						

9. *Question used with permission using a restricted licensing agreement that prohibits the reproduction of the item.*

10. *Question used with permission using a restricted licensing agreement that prohibits the reproduction of the item.*

11. Thinking back on this academic year (July 2019 to present), how satisfied were you with the following:

	Very Dissatisfied	Dissatisfied	Neutral	Satisfied	Very Satisfied
Your decision to become an emergency physician					
Your time for personal life (e.g., family, hobbies, dating, social life)					
Your ability to maintain healthy habits (e.g., regular exercise, eat healthy food, etc.)					
Your ability to complete regular health maintenance appointments (e.g., dental visits, PCP, OB/GYN)					

Suicidal Thoughts

12. During the past 12 months, have you had thoughts of taking your own life?
    a. Yes
    b. No

© 2021 Lall MD et al. *JAMA Network Open*.

This text should appear beneath question 12 on the same page.
Suicidal ideation should be taken seriously, and we urge you to seek medical attention. You can start by reaching out to your program director or calling the toll-free National Suicide Prevention Line (1-800-273-8255), available 24 hours a day, 7 days a week. The service is available to anyone. All calls are confidential. http://www.suicidepreventionlifeline.org.

© 2021 Lall MD et al. *JAMA Network Open*.
